# Amplicon sequencing data set of bacterial and phytoplankton communities during a record low-ice winter in the North American Great Lakes

**DOI:** 10.1128/mra.00369-26

**Published:** 2026-07-13

**Authors:** Neve C. Hudson, Katelyn M. Brown, Brian S. Gresham, Taylor E. Peace, James R. Zittel, Geoffrey M. DeLorie, Derek S. Niles, George S. Bullerjahn, Warren J. S. Currie, Steven A. Ruberg, R. Michael McKay

**Affiliations:** 1 Great Lakes Institute for Environmental Research, University of Windsor 8637, Windsor, Ontario, Canada; 2 CGC MORRO BAY (WTGB 106), U.S. Coast Guard Ninth District, Cleveland, Ohio, USA; 3 CGC NEAH BAY (WTGB 105), U.S. Coast Guard Ninth District, Cleveland, Ohio, USA; 4 Orange Force Marine Ltd., Port Stanley, Ontario, Canada; 5 Center for Great Lakes and Watershed Studies, Bowling Green State University 1888, Bowling Green, Ohio, USA; 6 Fisheries and Oceans Canada 6344, Burlington, Ontario, Canada; 7 NOAA Great Lakes Environmental Research Laboratory 53841, Ann Arbor, Michigan, USA; Nanchang University, Nanchang, Jiangxi, China

**Keywords:** Lake Erie, winter, ice, diatom, 16S, 18S

## Abstract

Bacterial and phytoplankton communities were profiled across a record low ice-cover winter (2024) and adjacent shoulder seasons in Lakes Erie, Huron, Superior, and St. Clair using 16S and 18S rRNA gene sequencing. Eukaryotic communities were dominated by diatoms and zooplankton, with Lake Superior exhibiting a distinct taxonomic composition.

## ANNOUNCEMENT

Lake Erie typically experiences extensive winter ice cover; however, ice extent across the Great Lakes is in decline and winter surveillance of planktonic communities remains limited (1, 2). An historic low ice winter in 2024 provided an opportunity to examine lower food web structure under conditions representative of a potential future ice-free state. Here, we used 16S and 18S rRNA gene sequencing to identify community structure during this predominantly ice-free period and compared it to conditions observed during a more typical ice climate experienced during winter 2025.

Biomass from water sampled using a Van Dorn bottle at 1 m depth was concentrated on Sterivex cartridge filters (0.22 µm; Millipore Corporation, Burlington, MA) and flash frozen in liquid nitrogen or dry ice. DNA was extracted using the DNeasy PowerWater kit (Qiagen, Germantown, MD) following manufacturer’s protocol with an additional heating step at 65 °C for 10 min following bead beating. Both 16S and 18S gene sequencing were performed using Earth Microbiome primers (3) and an Ion GeneStudio S5 Sequencer (Thermo Fisher Scientific, Waltham, MA) (Table 1). The runs had mean read lengths of 243 and 283. Libraries were prepared using the template preparation and sequencing described elsewhere using Ion510 & Ion520 & Ion530 kits for Ion Chef (Thermo Fisher Scientific) (4, 5). Raw sequences were filtered using the standard Ion Torrent Q15 criteria.

**TABLE 1:** Spatial and temporal metadata of sequenced samples collected from Great Lakes monitoring stations maintained by Canadian and U.S. federal agencies^*b*^

Sample	Date	Coordinates	No. of libraries	No. of raw 16S reads	No. of raw 18S reads	16S reads accession no.	18S reads accession no.
EC957	01/08/2024	41.6848 N 81.7421 W	3	48,278 33,872 44,714	61,654 50,926 48,314	SAMN55323522 SAMN55323654 SAMN55323683	SAMN55323740 SAMN55323872 SAMN55323901
EC958	01/08/2024	41.5259 N 81.7074 W	3	54,411 42,479 56,316	58,994 53,950 62,060	SAMN55323655 SAMN55323686 SAMN55323689	SAMN55323873 SAMN55323904 SAMN55323907
EC962	01/19/2025	41.715 N 82.178 W	3	10,154 11,081 45,003	38,733 15,380 23,610	SAMN55323697 SAMN55323701 SAMN55323722	SAMN55323915 SAMN55323919 SAMN55323934
EC967^*a*^	01/19/2025	41.892 N 82.664 W	3	30,873 30,965 16,193	15,287 26,477	SAMN55323690 SAMN55323721 SAMN55323723	SAMN55323908 SAMN55323935
EC962	01/22/2024	41.6707 N 82.0914 W	3	51,693 30,914 52,939	45,087 61,017 37,250	SAMN55323653 SAMN55323658 SAMN55323659	SAMN55323871 SAMN55323876 SAMN55323877
EC967	01/22/2024	41.8763 N 82.6018 W	3	78,592 59,483 74,667	49,881 58,381 52,091	SAMN55323521 SAMN55323651 SAMN55323665	SAMN55323739 SAMN55323869 SAMN55323883
EC962	01/31/2024	41.7167 N 82.1836 W	3	42,730 40,161 35,850	48,257 79,478 96,143	SAMN55323524 SAMN55323657 SAMN55323663	SAMN55323742 SAMN55323875 SAMN55323881
EC967	01/31/2024	41.8676 N 82.6639 W	3	39,270 30,062 31,948	37,437 53,073 47,750	SAMN55323523 SAMN55323649 SAMN55323664	SAMN55323741 SAMN55323867 SAMN55323882
EC966	02/03/2024	41.9814 N 82.6263 W	3	21,351 16,098 39,105	21,579 14,088 43,762	SAMN55323627 SAMN55323628 SAMN55323629	SAMN55323845 SAMN55323846 SAMN55323847
EC967	02/03/2024	41.8917 N 82.665 W	3	29,528 31,291 55,579	58,124 34,258 31,930	SAMN55323514 SAMN55323527 SAMN55323677	SAMN55323732 SAMN55323745 SAMN55323895
EC970	02/03/2024	41.8252 N 82.9752 W	3	25,179 24,498 30,242	55,295 49,596 18,805	SAMN55323512 SAMN55323672 SAMN55323678	SAMN55323730 SAMN55323890 SAMN55323896
EC971	02/03/2024	41.9388 N 83.0493 W	3	34,187 49,010 39,515	19,656 32,996 62,604	SAMN55323631 SAMN55323634 SAMN55323676	SAMN55323849 SAMN55323852 SAMN55323894
EC889	02/09/2024	42.1095 N 81.574 W	2	34,732 30,849	56,310 41,111	SAMN55323541 SAMN55323552	SAMN55323759 SAMN55323770
EC949	02/09/2024	42.24897 N 81.1088 W	2	48,027 25,077	29,838 43,300	SAMN55323667 SAMN55323673	SAMN55323885 SAMN55323891
EC953^*a*^	02/09/2024	42.207 N 81.442 W	2	31,300 16,951	31,059	SAMN55323619 SAMN55323620	SAMN55323837
EC961	02/09/2024	41.9077 N 82.1807 W	2	34,093 51,428	57,715 46,505	SAMN55323563 SAMN55323574	SAMN55323781 SAMN55323792
LSC-SP	02/09/2024	42.3381 N 82.9178 W	6	37,869 45,857 34,613 31,374 31,071 40,689	36,207 34,168 72,412 51,556 43,901 44,694	SAMN55323515 SAMN55323520 SAMN55323669 SAMN55323670 SAMN55323675 SAMN55323680	SAMN55323733 SAMN55323738 SAMN55323887 SAMN55323888 SAMN55323893 SAMN55323898
EC727	02/10/2024	42.816 N 79.3847 W	2	43,801 37,805	71,593 66,391	SAMN55323615 SAMN55323616	SAMN55323833 SAMN55323834
EC879	02/10/2024	42.5067 N 79.9 W	2	44,472 36,069	52,102 33,263	SAMN55323598 SAMN55323600	SAMN55323816 SAMN55323818
EC880	02/10/2024	41.935 N 81.656 W	2	41,267 29,029	41,216 45,940	SAMN55323519 SAMN55323530	SAMN55323737 SAMN55323748
EC887	02/10/2024	42.646 N 79.692 W	2	43,896 31,125	48,065 64,411	SAMN55323617 SAMN55323618	SAMN55323835 SAMN55323836
EC934	02/10/2024	42.7072 N 79.5112 W	2	50,310 36,671	33,804 49,479	SAMN55323586 SAMN55323597	SAMN55323804 SAMN55323815
EC939	02/10/2024	42.5693 N 79.99 W	2	38,863 39,058	40,262 28,480	SAMN55323621 SAMN55323623	SAMN55323839 SAMN55323841
EC945	02/10/2024	42.3988 N 80.642 W	2	31,920 79,098	41,346 33,077	SAMN55323517 SAMN55323674	SAMN55323735 SAMN55323892
LSC-MB	02/11/2025	42.457 N 82.421 W	3	11,096 83,24 32,114	24,281 17,793 21,179	SAMN55323692 SAMN55323694 SAMN55323724	SAMN55323910 SAMN55323912 SAMN55323936
EC966	02/18/2025	41.983 N 82.625 W	2	33,887 8,089	24,647 27,975	SAMN55323695 SAMN55323696	SAMN55323913 SAMN55323914
EC967	02/18/2025	41.892 N 82.667 W	1	26,171	8,672	SAMN55323720	SAMN55323932
EC958	02/20/2024	41.5153 N 81.7215 W	3	18,859 43,974 37,396	39,743 36,555 27,286	SAMN55323624 SAMN55323625 SAMN55323626	SAMN55323842 SAMN55323843 SAMN55323844
EC967	02/21/2025	41.891 N 82.666 W	4, 3	28,375 32,913 72,402 18,015	25,138 19,055 20,421	SAMN55323691 SAMN55323693 SAMN55323712 SAMN55323719	SAMN55323909 SAMN55323911 SAMN55323926
EC933	02/22/2024	42.8249 N 79.5676 W	2	38,338 24,207	35,785 122,317	SAMN55323646 SAMN55323681	SAMN55323864 SAMN55323899
EC944	02/22/2024	42.5334 N 80.6421 W	3	28,075 34,728 66,996	32,038 53,935 107,111	SAMN55323639 SAMN55323647 SAMN55323687	SAMN55323857 SAMN55323865 SAMN55323905
EC951	02/22/2024	42.4751 N 81.4415 W	2	73,260 68,341	85,812 50,233	SAMN55323644 SAMN55323688	SAMN55323862 SAMN55323906
EC881	02/23/2024	41.969 N 82.2084 W	3	37,755 45,387 42,539	27,595 34,470 49,337	SAMN55323609 SAMN55323648 SAMN55323684	SAMN55323827 SAMN55323866 SAMN55323902
EC888	02/23/2024	42.2812 N 81.6703 W	2	19,506 41,260	40,202 46,036	SAMN55323509 SAMN55323682	SAMN55323727 SAMN55323900
LSC-MB	02/27/2024	42.4689 N 82.411 W	2	36,286 54,263	26,651 26,995	SAMN55323630 SAMN55323632	SAMN55323848 SAMN55323850
EC971	03/04/2024	41.9498 N 83.0507 W	2	31,943 29,373	54,743 23,752	SAMN55323607 SAMN55323608	SAMN55323825 SAMN55323826
EC972	03/04/2024	41.8671 N 83.1981 W	2	30,638 31,132	20,538 35,317	SAMN55323635 SAMN55323636	SAMN55323853 SAMN55323854
EC970^*a*^	03/07/2024	41.8245 N 82.9765 W	3	32,369 37,588 32,424	31,281 35,676	SAMN55323511 SAMN55323613 SAMN55323638	SAMN55323729 SAMN55323856
EC947	03/07/2025	41.992 N 80.644 W	3, 2	11,094 127,674 24,301	37,572 34,633	SAMN55323702 SAMN55323715 SAMN55323716	SAMN55323920 SAMN55323929
EC955	03/07/2025	41.8 N 81.441 W	4, 3	41,120 138,776 66,593	48,536 45,298 66,567	SAMN55323707 SAMN55323709 SAMN55323717	SAMN55323924 SAMN55323925 SAMN55323930
EC888	03/11/2025	42.281 N 81.671 W	3	79,989 21,489 24,813	82,817 41,287 41,412	SAMN55323698 SAMN55323699 SAMN55323705	SAMN55323916 SAMN55323917 SAMN55323922
EC945	03/11/2025	42.4 N 80.642 W	3, 2	39,984 31,437 52,857	42,199 44,175	SAMN55323703 SAMN55323704 SAMN55323718	SAMN55323921 SAMN55323931
EC949	03/11/2025	42.257 N 81.106 W	3, 1	27,330 12,701 49,322	44,712	SAMN55323708 SAMN55323711 SAMN55323714	SAMN55323928
EC953	03/11/2025	42.208 N 81.441 W	3	17,143 138,128 34,560	55,040 72,745 80,135	SAMN55323700 SAMN55323706 SAMN55323713	SAMN55323918 SAMN55323923 SAMN55323927
EC1	03/12/2024	43.1542 N 82.4067 W	3	35,622 16,367 24,717	40,737 26,752 31,935	SAMN55323546 SAMN55323548 SAMN55323549	SAMN55323764 SAMN55323766 SAMN55323767
EC971	03/12/2024	41.9135 N 83.1068 W	3	19,465 38,610 51,050	29,124 39,037 31,492	SAMN55323542 SAMN55323544 SAMN55323550	SAMN55323760 SAMN55323762 SAMN55323768
EC54	03/19/2024	45.517 N 83.417 W	2	32,383 39,222	49,778 30,279	SAMN55323671 SAMN55323685	SAMN55323889 SAMN55323903
EC60	03/19/2024	45.902 N 83.518 W	2	41,759 30,944	N/A	SAMN55323610 SAMN55323612	N/A
EC69^*a*^	03/19/2024	46.078 N 84.028 W	3	20,625 31,030	25,530 10,241 35,766	SAMN55323614 SAMN55323637	SAMN55323728 SAMN55323832 SAMN55323855
EC341	03/21/2024	41.7987 N 82.2919 W	3	34,612 36,758 46,556	51,793 44,168 31,723	SAMN55323543 SAMN55323547 SAMN55323573	SAMN55323761 SAMN55323765 SAMN55323791
EC962	03/21/2024	41.7167 N 82.1832 W	3	40,047 31,265 32,373	31,597 26,666 43,353	SAMN55323538 SAMN55323545 SAMN55323571	SAMN55323756 SAMN55323763 SAMN55323789
EC967	03/21/2024	41.8764 N 82.6669 W	3	32,679 37,969 39,017	28,308 32,183 46,824	SAMN55323539 SAMN55323540 SAMN55323572	SAMN55323757 SAMN55323758 SAMN55323790
EC138	03/28/2024	48.417 N 88.933 W	3	74,964 35,078 36,186	31,350 21,156 22,184	SAMN55323508 SAMN55323589 SAMN55323679	SAMN55323726 SAMN55323807 SAMN55323897
EC12	03/29/2024	47.037 N 85.03 W	3	35,931 35,717 44,645	41,850 33,116 40,346	SAMN55323585 SAMN55323633 SAMN55323645	SAMN55323803 SAMN55323851 SAMN55323863
EC2	03/29/2024	46.543 N 84.748 W	3	42,626 31,737 32,906	23,045 22,328 19,666	SAMN55323584 SAMN55323611 SAMN55323622	SAMN55323802 SAMN55323829 SAMN55323840
EC23	03/29/2024	47.213 N 85.633 W	3	42,374 22,675 26,005	26,002 14,068 22,894	SAMN55323507 SAMN55323581 SAMN55323599	SAMN55323725 SAMN55323799 SAMN55323817
EC5	03/29/2024	46.733 N 84.793 W	3	42,861 35,768 35,883	26,342 28,731 25,185	SAMN55323591 SAMN55323656 SAMN55323668	SAMN55323809 SAMN55323874 SAMN55323886
EC1238	04/15/2024	42.6325 N 81.2101 W	2	37,318 82,140	25,198 34,726	SAMN55323565 SAMN55323566	SAMN55323783 SAMN55323784
EC1239	04/15/2024	42.615 N 81.2091 W	3	20,562 37,396 36,912	35,319 36,392 29,488	SAMN55323567 SAMN55323587 SAMN55323596	SAMN55323785 SAMN55323805 SAMN55323814
EC1240	04/15/2024	42.5703 N 81.2033 W	3	44,965 42,675 39,522	20,055 39,749 18,155	SAMN55323579 SAMN55323588 SAMN55323592	SAMN55323797 SAMN55323806 SAMN55323810
ER30	04/15/2024	42.4297 N 81.2045 W	3	44,539 42,004 57,078	24,584 55,226 45,618	SAMN55323569 SAMN55323570 SAMN55323580	SAMN55323787 SAMN55323788 SAMN55323798
ER31	04/15/2024	42.2531 N 81.1067 W	3	58,314 47,899 30,846	43,396 32,288 21,399	SAMN55323568 SAMN55323582 SAMN55323594	SAMN55323786 SAMN55323800 SAMN55323812
100-G-3	04/22/2024	42.5583 N 80.975 W	3	33,507 32,013 35,099	37,339 35,795 22,689	SAMN55323537 SAMN55323577 SAMN55323590	SAMN55323755 SAMN55323795 SAMN55323808
100-I-2	04/22/2024	42.5664 N 80.9481 W	3	29,646 58,347 33,203	31,993 19,841 28,653	SAMN55323578 SAMN55323583 SAMN55323595	SAMN55323796 SAMN55323801 SAMN55323813
LSC-MB	05/14/2024	42.4689 N 82.4106 W	2	97,630 38,405	27,977 37,806	SAMN55323575 SAMN55323576	SAMN55323793 SAMN55323794
ER30	10/23/2023	42.4302 N 81.205 W	4	9,494 36,388 38,881 33,574	28,486 33,929 31,887 54,916	SAMN55323516 SAMN55323528 SAMN55323642 SAMN55323643	SAMN55323734 SAMN55323746 SAMN55323860 SAMN55323861
ER31^*a*^	10/23/2023	42.2535 N 81.1065 W	4	66,183 65,940 56,737 37,285	57,081 55,823 33,647	SAMN55323529 SAMN55323531 SAMN55323604 SAMN55323605	SAMN55323747 SAMN55323749 SAMN55323823
OA1^*a*^	10/23/2023	42.5186 N 81.1961 W	4	59,614 46,189 26,944 34,057	42,203 37,831 25,808	SAMN55323513 SAMN55323525 SAMN55323535 SAMN55323603	SAMN55323731 SAMN55323743 SAMN55323753
OA2	10/23/2023	42.57195 N 81.2026 W	4	40,344 22,552 24,772 24,988	60,652 52,876 20,647 24,193	SAMN55323534 SAMN55323536 SAMN55323640 SAMN55323641	SAMN55323752 SAMN55323754 SAMN55323858 SAMN55323859
OA3^*a*^	10/23/2023	42.6178 N 81.2066 W	4	32,971 39,338 36,711 43,359	56,061 28,834	SAMN55323526 SAMN55323532 SAMN55323601 SAMN55323602	SAMN55323744 SAMN55323750
EC1240	11/02/2024	42.57 N 81.203 W	3	41,595 46,160 21,897	46,615 39,811 35,718	SAMN55323554 SAMN55323555 SAMN55323558	SAMN55323772 SAMN55323773 SAMN55323776
ER30	11/02/2024	42.43 N 81.205 W	3	20,591 37,046 29,963	37,500 40,443 30,713	SAMN55323553 SAMN55323557 SAMN55323564	SAMN55323771 SAMN55323775 SAMN55323782
ER31	11/02/2024	42.254 N 81.107 W	3	36,900 27,358 53,133	19,402 48,953 21,338	SAMN55323556 SAMN55323559 SAMN55323560	SAMN55323774 SAMN55323777 SAMN55323778
RAS	11/02/2024	41.999 N 81.588 W	4	46,912 47,309 59,417 14,459	61,428 54,047 36,433 24,419	SAMN55323551 SAMN55323561 SAMN55323562 SAMN55323593	SAMN55323769 SAMN55323779 SAMN55323780 SAMN55323811
LSC-SP	11/27/2023	42.3381 N 82.9178 W	3	35,205 26,793	28,183 44,142	SAMN55323518 SAMN55323533	SAMN55323736 SAMN55323751
EC957	12/04/2023	41.6826 N 81.7441 W	4	30,113 40,804 42,652 24,037	44,761 49,491 41,693 28,357	SAMN55323650 SAMN55323652 SAMN55323662 SAMN55323666	SAMN55323868 SAMN55323870 SAMN55323880 SAMN55323884
EC958	12/04/2023	41.5259 N 81.7074 W	2	28,605 27,577	29,015 24,889	SAMN55323660 SAMN55323661	SAMN55323878 SAMN55323879

^*a*^ Variation in the number of libraries is due to a failed replicate. Samples with a removed replicate (< 5000 reads) are indicated.

^*b*^ N/A, not applicable.

Sample sequences were processed in RStudio (v.4.5.1) with package dada2 (v.1.36.0). Default parameters of all packages were used, and the standard dada2 v.1.8 tutorial was followed unless otherwise specified (6–9). Both 16S and 18S samples were sequenced and processed in two sets and then merged following dereplication. Sequences were trimmed to remove low-quality bases and primers for an average quality score of 30 (16S: trimLeft = 50, truncLen = 325, maxLen = 350; 18S: trimLeft = 46, truncLen = 200, maxLen = 225). Reads were dereplicated into amplicon sequence variants (ASVs), denoised (homopolymer gap penalty = −1, band size = 32), error-corrected, and chimeras were removed. Approximately 50% of reads for 16S and 18S gene sequences remained. 16S taxonomy was assigned using SILVA v.138.2 and Protist Ribosomal Reference v.5.1.0 for 18S, using the naïve Bayesian classifier method from dada2 (7). Samples with less than 5,000 remaining quality-controlled reads were discarded. ASVs of replicate samples were summed by site and date, and classified 16S chloroplast and mitochondrial ASVs were removed before passing through phyloseq (v.1.52.0) to calculate relative abundance. Plots were generated using tidyverse (v.2.0.0), ggh4x (v.0.3.1), and RColorBrewer (v.1.1-3) (10–13).

**Fig 1:**
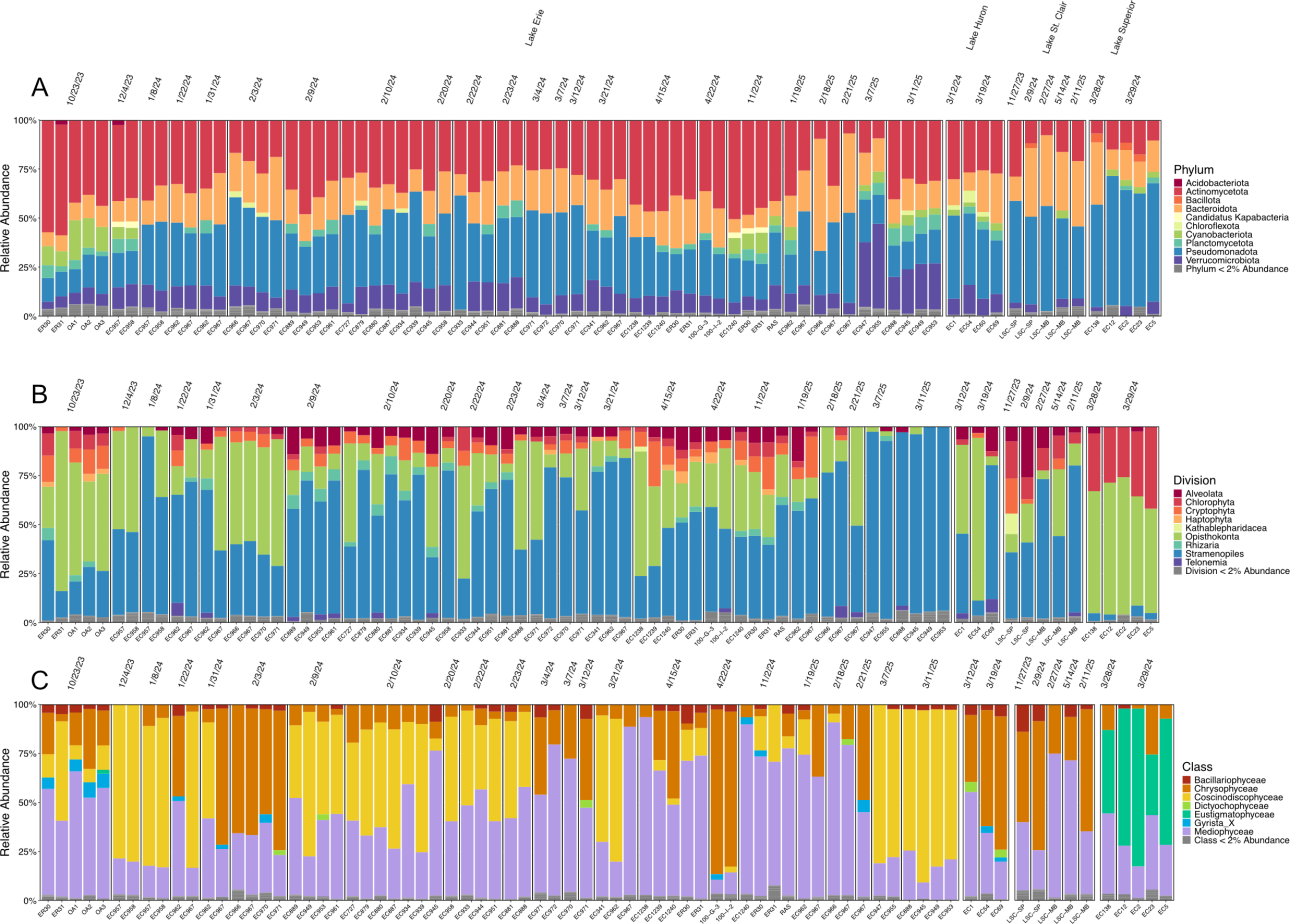
Relative abundance of (**A**) classified bacterial phyla, (**B**) classified eukaryotic divisions, and (**C**) classified Stramenopile classes in the Great Lakes (Erie, Huron, Superior, and St. Clair) during the predominantly ice-free winter of 2024 and the ice-covered winter of 2025. 18S reads classified to the domain Bacteria were removed, and a minimum abundance filter was applied to the 16S Phyla and 18S Divisions, to group all ASVs with <2% abundance. The Division Stramenopile was subset and identified at the Class level.

The prokaryotic community was consistently dominated by Actinomycota, Pseudomonadota, and Bacteroidota (Fig. 1A). Among the lakes, Lake Superior exhibited a distinct eukaryotic composition consisting of ~70% Opisthokonta and ~25% Alveolata (Fig. 1B), with *Dictyochophyceae* as the dominant Stramenopile class (Fig. 1C). In contrast, Lakes Erie, Huron, and St. Clair were dominated by Stramenopiles and Opisthokonta (Fig. 1B). Within Stramenopiles, classes *Mediophyceae*, *Coscinodiscophyceae*, and *Chrysophyceae* were abundant in Lake Erie, whereas *Coscinodiscophyceae* was largely absent from Lakes Huron and St. Clair (Fig. 1C).

## Data Availability

The raw reads from this amplicon sequencing project have been deposited in NCBI GenBank under BioProject accession PRJNA1422841. Environmental metadata can be accessed from the Winter survey data from Lake Erie published in the BCO-DMO repository (14, 15).
